# An active *Catharanthus roseus* desacetoxyvindoline-4-hydroxylase-like gene and its transcriptional regulatory profile

**DOI:** 10.1186/1999-3110-55-29

**Published:** 2014-03-13

**Authors:** Chen Zhou, Jin Zhang, Shu-Juan Zhao, Zhi-Bi Hu

**Affiliations:** The SATCM Key Laboratory for New Resources & Quality Evaluation of Chinese Medicines and Shanghai Key Laboratory of Complex Prescription, Institute of Traditional Chinese Material Medica, Shanghai University of Traditional Chinese Medicine, 201203 Shanghai, China

**Keywords:** *Catharanthus roseus*, C_20_hi cells, Desacetoxyvindoline-4-hydroxylase, d4h-like, GUS activity, Real-time quantitative PCR, Transient expression

## Abstract

**Background:**

Desacetoxyvindoline-4-hydroxylase is a key enzyme in the biosynthesis of vindoline, the important intermediate leading to vinblastine and vincristine in *Catharanthus roseus*.

**Results:**

A *d4h-like* gene has been isolated from *C. roseus* C_20_hi cells based on an EST sequence from the Suppression Subtractive Hybridization cDNA library. The full length cDNA of *d4h-like* was 1427 bp encoding 372 amino acids. It had 66% identities and 80% positives with *d4h* at the amino acid level. It belonged to 2-oxoglutarate dependent oxygenase superfamily as *d4h* did. Real-time quantitative PCR analysis revealed that *d4h-like* was expressed high in roots, flowers and C_20_hi cells, very low in leaves and stems. Methyl jasmonate could significantly increase the accumulation of *d4h-like* transcripts. 2,4-D inhibited its expression. An approximate 2,910 bp of 5′-promoter region of *d4h-like* was obtained, fused to *GUS* reporter gene and analyzed with fluorescence quantitative assays using transient expression in *C. roseus* cell suspensions, indicating that *d4h-like* promoter could drive GUS gene expression in vivo.

**Conclusion:**

These results suggest that *d4h-like* is closely related with *d4h* in the genetic evolution but with different transcriptional expression profiles. It may be revolved in the hormone-independency of C_20_hi cells.

**Electronic supplementary material:**

The online version of this article (doi:10.1186/1999-3110-55-29) contains supplementary material, which is available to authorized users.

## Background

Vinblastine and vincristine are powerful anticancer compounds produced by Madagascar periwinkle (*Catharanthus roseus* (L.) G. Don.) and derived by the coupling of the monomeric alkaloids catharanthine and vindoline in plants (El-Sayed and Verpoorte 2007). Research revealed that vindoline was transformed from tabersonine through a sequence of six strictly ordered enzyme reactions including aromatic hydroxylation, *O*-methylation, hydration of the 2,3-double bond, *N*(1)-methylation, 4-hydroxylation, and 4-*O*-acetylation (El-Sayed and Verpoorte 2007). Desacetoxyvindoline-4-hydroxylase (D4H, E.C.1.14.11.11) catalyzed the 4-hydroxylation of desacetoxyvindoline to form deacetylvindoline (Vazquez-Flota et al. 1997) which consequently converted to vindoline by deacetylvindoline-4-O-acetyltransferase (DAT) (Power et al. 1990). D4H was a 2-oxoglutarate-dependent dioxygenase and could be induced by light and red-light (Verpoorte et al. 2000). It was localized at cytoplasm and its mRNA were associated with the laticifer and idioblast cells of the leaves, stems and flower buds (St-Pierre et al. 1999). Hydroxylase assays and RNA-blot hybridization studies showed that D4H activity was closely related to the levels of *d4h* transcripts, occurring predominantly in young leaves and in much lower levels in stems and fruits (Vazquez-Flota et al. 1997). The biosynthesis of vindoline was also light-induced and had tissue-specific accumulation profile which was coincided with the expression patterns of *d4h* (El-Sayed and Verpoorte 2007).

The D4H enzyme and corresponding gene were elucidated in *C. roseus*. Till now, there was no homologous gene of *d4h* having been reported. Recently, a special *C. roseus* cell line C_20_hi was achieved in culturing the *C. roseus* C_20_D cells by gradually reducing the hormones in the media in our lab. These cells were hormone-independent and could produce much more tryptamine, serpentine, and ajmalicine than the original cell line C_20_D. Using cell lines C_20_hi and C_20_D, a Suppression Subtraction Hybridization (SSH) cDNA library was established. Interestingly, a 671 bp fragment, containing the 3′-untranslated region, highly similar to *d4h* at the amino acid level was obtained in the library. Here, we reported the isolation and characterization of the full length cDNA and the promoter regions of *d4h-like* (GenBank accession number: EF640810, GU363550).

## Methods

### Plant and cell material

*Catharanthus roseus* cells were cultured in liquid B5 basic media by adding 2,4-D (2,4-dichlorophen-oxyacetic acid) and kinetin for C_20_, 2,4-D for C_20_D, and without any hormone for C_20_hi with shaking at 100 rpm in a shaker at 25°C under dark. *C. roseus* plant was cultivated in the growth chamber at 25°C with a photoperiod of 16 h light and 8 h dark.

### RNA isolation

Total RNAs were isolated using RNA Extraction Kit (Takara Biotechnology, Dalian, China) from different tissues and cells of *C. roseus* and checked with agarose gel electrophoresis and spectrophotometer (HITACHI U-2900) analysis. RNA samples were stored at −80°C before use.

### Cloning of the full length cDNA, genomic DNA and promoter region of *d4h-like*

The 5′-RACE was performed using SMART RACE cDNA Amplification Kit (Clontech). Gene-specific primers were designed based on the 671 bp of EST sequence of *d4h-like* from SSH cDNA library. Universal Primer A Mix (UPM) coupled with 5′ nested universal primer (NUP) were used to do the PCR amplification according to the direction. PCR products were recovered from 1% agarose gel, inserted into pMD18-T vector (Takara), and sequenced. Full length cDNA of *d4h-like* was amplified using gene-specific primers D4HL-F and D4HL-R.

GenomeWalker^TM^ Universal Kit (Clontech) was used to isolate the 5′- upstream sequence of *d4h-like*. DNA was isolated with DNA Extraction Kit (Takara) from *C. roseus* leave. Genomic DNA of *d4h-like* was cloned using gene-specific primers. To get the 5′-promoter region, DNA was digested with restriction enzymes *Dra* I, *EcoR* V, *Pvu* II, and *Stu* I, purified and ligated with GenomeWalker Adaptor, separately. Primary and secondary PCR were consequentially carried out. PCR products were ligated into T-vector and sequenced as described above. All the primers used in this research were listed in Additional file 1: Table S1.

### Sequence analysis

Sequence analysis and multiple alignments were conducted using Vector NTI 7.1 (InforMax, USA). Putative *cis-* regulatory elements of the promoter region were predicted with PlantCARE online software. Phylogenetic tree was established applying MegAlign with CLUSTAL W method.

### Protein expression in *Escherichia coli*

Open reading frame fragment of *d4h-like* cDNA amplified with high-fidelity Taq DNA polymerase (TaKaRa) was fused into the pRSET (Invitrogen) vector and confirmed by sequencing. The vector was transferred into *E. coli* strain BL21 (DE3) to express the proteins according to the direction for pRSET kit. Water-soluble recombinant proteins were separated with SDS-PAGE electrophoresis.

### Real-time quantitative PCR and semi-quantitative RT-PCR analysis

About 2 μg of total RNA was used as template to perform reverse transcription with PrimeScript RT Reagent Kit (TaKaRa). Real-time quantitative PCR (qPCR) was carried out on an ABI StepOnePlus PCR System (Applied Biosystems) using the SYBR premix ExTaq polymerase following thermal cycling conditions recommended by the manufacturer. RSP9 was used as internal reference gene (Peebles et al. 2009). Each sample was repeated at least three times. Data were analyzed with StepOne software V2.1.

The semi-quantitative RT-PCR was performed with β-actin as the reference control. The PCR program was as followed: 94°C for 2 min, followed by 32 cycles (for *d4h-like* gene) or 28 cycles (for *β-actin* gene) at 94°C for 15 s, 53°C for 20 s and 72°C for 60 s, and then a final extension at 72°C for 5 min. PCR products were separated on 1% agarose gels stained with ethidium bromide (10 mg/mL).

### Construction of promoter-GUS fusions

A modified pCaMV35S: GUS vector was created by replacing GFP in pCAMBIA1302 (http://www.cambia.org) with a *GUS* gene. Promoter: GUS vectors were generated by replacing the CaMV35S promoter cassette with promoter fragments, −1845/+140 bp, −1167/+140 bp, and −483/+140 bp, respectively. Each fusion was verified with DNA-sequencing. Plasmid was introduced into *A. tumefaciens* GV3101 through triparental mating using *Escheridhia coli* harboring pRK 2013 as conjugal helper strain.

### *Agrobacterium*-mediated transient expression

Transient expression was performed according to Wang et al. (2010). Briefly, individual colony of transformed *A. tumefaciens* was picked and cultured to a final OD_600_ of 0.6 in Luria–Bertani liquid media containing kanamycin (100 mg/L) and rifampicin (100 mg/L). *A. tumefaciens* pellets were collected, washed and added into flasks containing 20 mL of fresh *C. roseus* C_20_hi cells and 100 μM of acetosyringone. After cultivation under 28°C for another 48 h, cells were collected to do the expression assay. Cells infected with either *A. tumefaciens* containing pCaMV35S: GUS or *A. tumefaciens* only was used as positive and negative control, respectively. Each assay was performed triplicate.

### GUS activity assay

GUS activity was measured according to Jefferson and Kavanagh (1987). *C. roseus* transient transgenic cells were homogenized in protein extraction buffer (0.05 M NaPO_4_, pH 7.0, 0.1% SDS, 10 mM EDTA, 20% methanol, 10 mM β-ME, Triton X-100) and centrifuged. Supernatant were used to do the assay with fluorometer. Protein concentration was determined using Bradford method (Bio-Rad, USA). GUS enzyme activity was expressed as nanomolar of 4-MU produced per minute per milligram protein.

## Results and discussion

### Cloning and characteristics of *d4h-like* cDNA and genomic DNA

**Figure 1:**
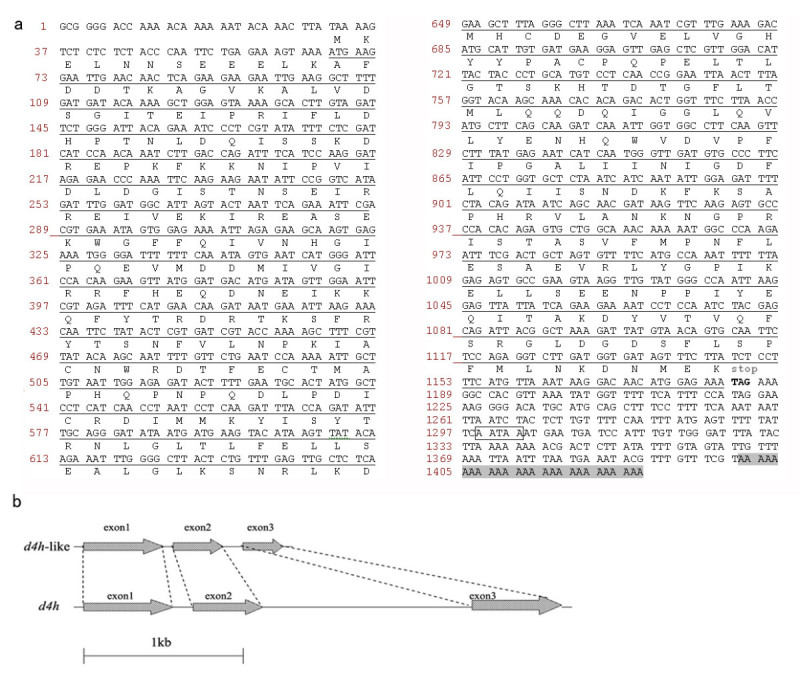
**cDNA sequence and genomic structute of** ***d4h-like*** **a) Nucleotide sequence of** ***d4h-like*** **cDNA and the predicted amino acid residues.** The open reading frame was underlined. Corresponding amino acid residues were above the nucleotide sequence. Putative polyadenylation signal was in box and the polyA tail was highlighted with shadow. **b)** Genomic organization of *d4h-* like and *d4h*.

**Figure 2:**
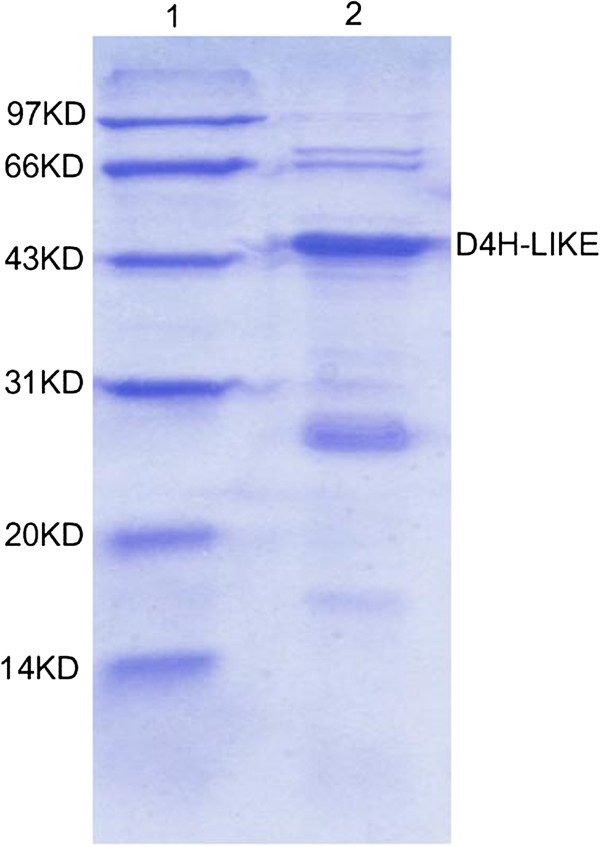
**SDS-PAGE result of D4H-LIKE recombinant protein.** Lane 1: Protein molecular weight marker. Lane 2: Purified water-soluble recombinant D4H-LIKE protein.

A 910 bp of PCR product was obtained applying 5′-RACE method. The full-length cDNA of *d4h-like* (Figure 1a) was 1427 bp encoding 372 amino acids with a calculated molecular mass of 42.7 kDa and isolectric point (pI) of 5.31. The ORF of *d4h-like* was fused with the 33 amino acid residues in the pRSET vector at the N-terminal. After induced by IPTG, an approximate 44KD of specific water-soluble protein was expressed in *E. coli* BL21 (Figure 2), which was right the predicted molecular weight of the recombinant D4H-LIKE protein. The 5′-untranslated region (UTR) was 68 bp and the 3′-UTR was 240 bp including 26 base poly(A)^+^ tail. Analysis with tBlastX showed that *d4h-like* had 66% (250/378) identities and 80% (303/378) positives to *d4h* at the amino acid level.

The genomic organization of *d4h-like* was similar with *d4h* (Figure 1b). It contained three exons and two introns, 85 and 128 bp in length, being located at the same conserved amino acid consensus sequences as those of *d4h*. But either intron of *d4h-like* was shorter than the corresponding one of *d4h*, which was 205 and 1720 bp.

### Alignment of D4H-LIKE with the other dioxygenases

**Figure 3:**
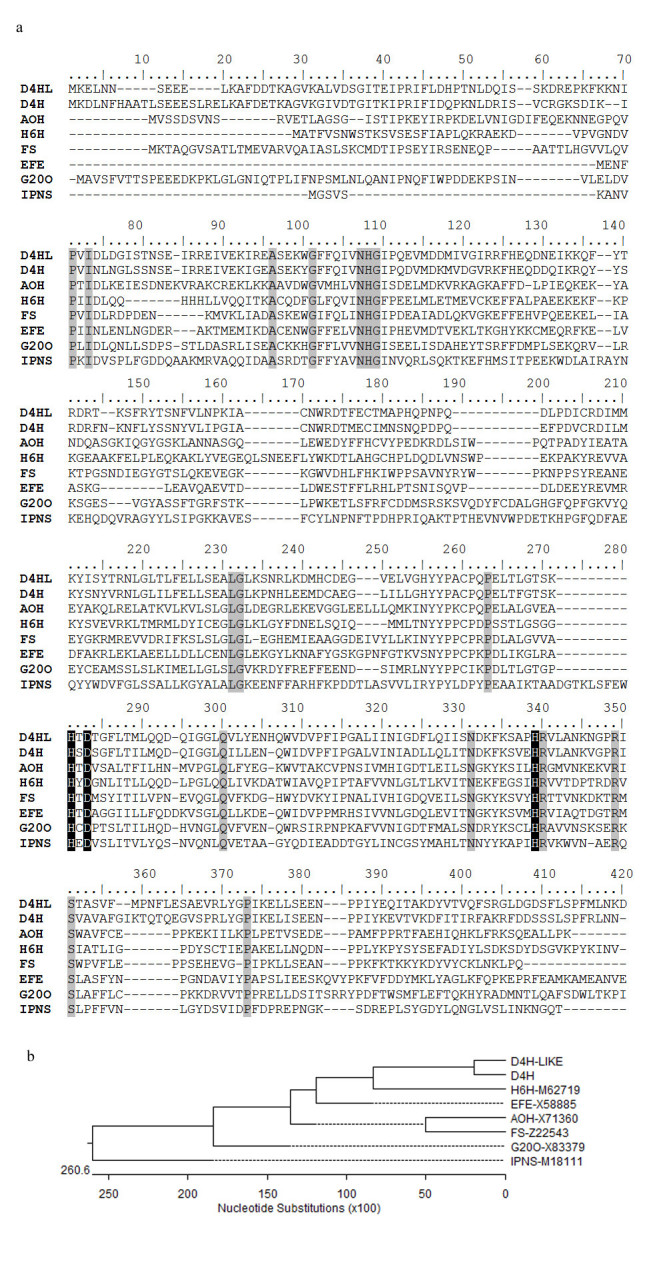
**Relationship of** ***d4h-like*** **with other oxygenase genes. a)** Alignment of D4H-like with other dioxygenases. D4H, desacetoxyvindoline-4-hydroxylase (GenBank accession number: U71604); AOH, anthocyanidin synthase from *Anthirrhinum candida* (GenBank accession number: X71360); H6H, hyoscyamine 6-hydroxylase from *Hyoscyamus niger* (GenBank accession number: M62719); FS, flavonol synthase from *Petunia hybrida* (GenBank accession number: Z22543); EFE, ethylene forming enzyme from tomato (GenBank accession number: X58885); G20O, gibberellin 20-oxidase from *Arabidopsis thaliana* (GenBank accession number: X83379); IPNS, isopenicillin N-synthase from *Aspergillus nidulans* (GenBank accession number: M18111). The 19 conserved residues were highlighted in shadow boxes and the binding site for ferrous ions was marked with white characters in black boxes. **b)** Phylogenetic tree constructed with D4H-like and other 2-oxoglutarate dependent oxygenases using MegAlign software.

NCBI conserved domain-searching results using Swissprot database indicated that the identity of D4H-LIKE with the other dioxygenases (Figure 3a) ranked from 27% for IPNS from *Aspergillus nidulans* (Ramon et al. 1987), 29% for Hyoscyamine 6-beta-hydroxylase (H6H) from *Hyoscyamus niger* (Matsuda et al. 1991) and anthocyanidin synthase (AS) from apple (Davis 1993), 33% for Ethylene-forming enzyme from *tomato* (Holdsworth et al. 1987) and G20O from *Arabidopsis thaliana* (Phillips et al. 1995) to 34% for Flavonol synthase (FS) from *Petunia x hybrida* (Holton et al. 1993). These enzymes and D4H all belonged to 2OG-Fe(II) oxygenase superfamily including 2-oxoglutarate (2OG) and Fe(II)-dependent oxygenase members. The C-terminal of this superfamily enzymes contained a prolyl 4-hydroxylase alpha subunit and the holoenzyme had the activity of procollagen-proline dioxygenase (EC:1.14.11.2) catalyzing the following reaction: L-proline-[procollagen] + 2-oxoglutarate + O(2) = trans-4-hydroxy-L- proline-[procollagen] + succinate + CO(2) (Helaakoski et al. 1995). Among the 19 highly conserved residues including Ala_84_, Gly_89_, Gly_97_, His_96_, His_235_, His_292_, Pro_226_, Leu_197_, Gln_253_, Arg_293_, and Ser_304_, the triad His_235_-X-Asp_237,_ together with His_292_ (Figure 3a), were proved to be the binding sites for ferrous ions in IPNS (Roach et al. 1995). The equivalent residues, highlighted with white characters in black boxes in Figure 3a, were also conserved in D4H-LIKE and D4H. All the information indicates that D4H-LIKE has the characteristics of oxidase, in particular, the enzymatic characteristics associated with Fe(II)-dependent oxygenase just like D4H.

### Phylogenetic analysis

The phylogenetic tree (Figure 3b) established with MegAlign software (DNASTAR Inc., Madison, WI, USA) showed that D4H-LIKE was closely clustered with D4H and kept a little far from EFE, AOH, FS, G20O, and IPNS. This result, taken together with the similar gene structure, implied that D4H-LIKE and D4H were closely related with each other in the genetic evolutionary process and D4H-LIKE may have similar functions in *C. roseus*.

### Isolation and characterization of *d4h-like* promoter

An approximately 2,910 bp promoter region of *d4h-like* was isolated. The promoter contained a typical TATA box, present at -25 bp upstream of the transcriptional start site (TSS). Analysis with PlantCARE showed that a number of potential *cis-* acting elements corresponding to regulatory signals existed upstream of the TATA-box in this region (Table 1 and Additional file 1: Figure S1). AT-rich sequence was element for maximal elicitor-mediated activation. HSE was related to heat stress responsiveness. ARE was *cis-* acting regulatory element essential for the anaerobic induction. TGACG-motif involved in the MeJA-responsiveness. GARE-motif was involved in the gibberellin responsiveness and ERE acted as ethylene-responsive element. The existence of such *cis-* acting elements in *d4h-like* promoter indicated that *d4h-like* might be controlled by a complicated regulatory mechanism and probably responded to various developmental and environmental signals.

**Table 1:** **Putative cis-elements in** ***d4h-like*** **promoter**

Motif name	Sequence	Position	Motif numbers	Function
O2-site	GATGATGTGG	−2858/-2849(−)	1	Cis-acting regulatory element involved in zein metabolism regulation
TC-rich repeats	GTTTTCTTAC	−2422/-2413(+)	2	Cis-acting element involved in defense and stress responsiveness
		−1254/-1245(+)		
Sp1	CC(G/A)CCC	−2463/-2458(−)	2	Light responsive element
		−2369/-2364(+)		
HSE	AAAAAATTTC	−2307/-2298(+)	2	Cis-acting element involved in heat stress responsiveness
		−359/-350(+)		
Box 4	ATTAAT	−2236/-2231(+)	4	Part of a conserved DNA module involved in light responsiveness
		−1677/-1672(+)		
		−800/-795(+)		
		−236/-231(+)		
TCCC-motif	TCTCCCT	−2253/-2247(+)	1	Part of a light responsive element
ARE	TGGTTT	−2207/-2202(+)	3	Cis-acting regulatory element essential for the anaerobic induction
		−2092/-2087(+)		
		−186/-181(−)		
TGACG-motif	TGACG	−2167/-2163(+)	2	Cis-acting regulatory element involved in the MeJA-responsiveness
		−1180/-1176(+)		
Box I	TTTCAAA	−1980/-1974(+)	4	Light responsive element
		−1411/-1405(+)		
		−380/-374(+)		
		−914/-908(−)		
I-box	AAGATAAGA	−1965/-1957(−)	3	Part of a light responsive element
		−1140/-1132(−)		
		−1085/-1077(−)		
Box II	TGGTAATAA	−1251/-1243(−)	1	Part of a light responsive element
ERE	ATTTCAAA	−1786/-1779(−)	2	Ethylene-responsive element
		−381/-374(+)		
Box III	CATTTACACT	−1650/-1642(+)	1	Protein binding site
GA-motif	ATAGATAA	−1557/-1550(+)	2	Part of a light responsive element
		−671/-664(+)		
AT-rich sequence	TAAAATACT	−1419/-1411(+)	1	Element for maximal elicitor-mediated activation (2copies)
TCT-motif	TCTTAC	−1338/-1333(+)	1	Part of a light responsive element
GARE-motif	AAACAGA	−892/-886(−)	1	Gibberellin-responsive element
GAG-motif	AGAGAGT	−286/-280(−)	1	Part of a light responsive element
G-box	CACATGG	−227/-221(−)	1	Cis-acting regulatory element involved in light responsiveness
circadian	CAANNNNATC	−156/-146(+)	1	Cis-acting regulatory element involved in circadian control
TATA-box	TATA(T/A)A(T/A)A	−25/-18(+)	1	Core promoter element around −30 of transcription start

### Expression profile of *d4h-like* in *C. roseus* plant and the cultured cells

**Figure 4:**
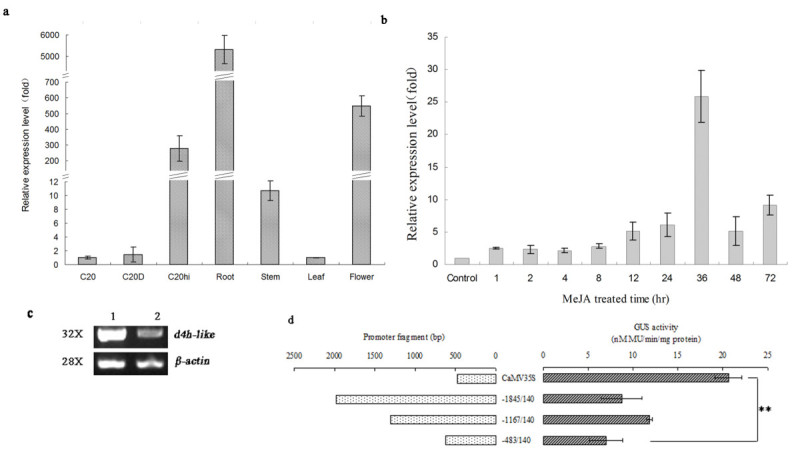
**Transcriptional analysis of** ***d4h-like*** **gene. a)** Expression profile of *d4h-like* in *C. roseus* plant and suspension cells. RSP9 was used as the reference control. **b)** MeJA effect on the *d4h-like* transcription. C_20_hi cells cultured for 14 d just before adding MeJA were used as control. **c)** Semi-quantitative RT-PCR result of *d4h-like* by addition of 2,4-D into the media. 1: C_20_hi cells in B5 basic media; 2:C_20_hi cells in B5 media adding of 2,4-D; “32×” and “28×” meant the PCR amplification cycle was 32 and 28, respectively. **d)** GUS transient expression assay. Left panel represented CaMV35S and 5′-deletion *d4h-like* promoter fragments constructions. Right panel displayed the corresponding GUS activities. Double stars indicated the difference between the two samples was significant. Each sample was triplicates.

RT-qPCR analysis revealed the transcripts of *d4h-like* was accumulated high in roots and flowers, and very low in stems and leaves (Figure 4a). The expression level of *d4h-like* in roots was about 100-fold, 530-fold, and 5300-fold higher than that in flowers, stems, and leaves, respectively. Among the three cell lines, C_20_hi cells exhibited the highest capacity to accumulate *d4h-like* mRNA. Both C_20_D and C_20_ cells accumulated very little *d4h-like* transcripts whose level was about 200-fold and 270-fold lower than that in C_20_hi, respectively (Figure 4a). This result suggested that *d4h-like* has root-specific expression characteristics.

### Effects of MeJA and 2,4-D on the transcript accumulation of *d4h-like*

After treating with MeJA, the transcription of *d4h-like* was gradually increased, reaching the highest level, about 25-fold high compared with the control at the 36^th^ hr, and then decreased but still at an up-regulated level after 48 and 72 hr (Figure 4b), suggesting that MeJA could induce *d4h-like* to express. Since the EST fragment of *d4h-like* was obtained through screening the SSH cDNA library established using C_20_hi and C_20_D cells and the difference between these two cell lines was that C_20_hi grew well in B5 media without 2,4-D, we speculate that the high transcription of *d4h-like* in C_20_hi cells may result from the absence of 2,4-D in the media. To demonstrate this speculation, 2 mg/L of 2,4-D was added into B5 media culturing C_20_hi cells and the transcripts of *d4h-like* was detected with semi-quantitative RT-PCR, which proved the expression of *d4h-like* was obviously inhibited by 2,4-D (Figure 4c).

Taking together with the existence of ethylene-, gibberellin-, MeJA-responsive elements and circadian control regulatory element in the promoter region, we suggest that *d4h-like* may be involved in the growth of C_20_hi cells.

### Function analysis of the *d4h-like* promoter in *C. roseus* suspension cells

Transient expression assay revealed that the activity of *d4h-like* promoter was 8.77 ± 2.29 nM MU/min/mg proteins for −1845/+140 bp, 11.85 ± 0.30 nM MU/min/mg proteins for −1167/+140 bp, and 7.00 ± 1.84 nM MU/min/mg proteins for −483/+140 bp fragment, and that of the CaMV35S was 20.65 ± 1.494 nM MU/min/mg proteins (Figure 4d). Analysis with SPSS19.0 revealed that there were no significant difference between the 35S promoter and the −1845/+140 bp fragment (*p* = 0.512 > 0.05), and the −1167/+140 bp fragment (*p* = 0.316 > 0.05), which indicated that the −1167/+140 bp region had the capability to drive GUS to express in transiently transformed *C. roseus* cells. The −483/+140 bp fragment showed significant difference with the 35S promoter (*p* = 0.020 < 0.05), implying that the *cis-* acting elements within −1167/-483 bp region may be necessary for the transcription of the gene. All the results suggested that *d4h-like* was an active gene in vivo.

## Conclusion

In general, an active *d4h-like* gene was cloned from *C. roseus* which showed different transcriptional expression profiles from *d4h*. It will be interesting to investigate the roles and the relationship between *d4h-like* and the biosynthesis of alkaloids such as vindoline, tryptamine, serpentine, and ajmalicine in *C. roseus*.

## Electronic supplementary material

Additional file 1: Table S1: Primers used for PCR. **Figure S1.** Analysis of *d4h-like* promoter with PlantCare. (DOC 796 KB)

Below are the links to the authors’ original submitted files for images.

Authors’ original file for figure 1

Authors’ original file for figure 2

Authors’ original file for figure 3

Authors’ original file for figure 4
