# Supplementary material for: An active Catharanthus roseus desacetoxyvindoline-4-hydroxylase-like gene and its transcriptional regulatory profile
Source: Bot Stud. 2014 Mar 13;55:29. doi: 10.1186/1999-3110-55-29 (PMC5430314; doi:10.1186/1999-3110-55-29)
Supplement: Supplementary file 1 — Additional file 1: Table S1: Primers used for PCR. Figure S1. Analysis of d4h-like promoter with PlantCare. (DOC 796 KB) [file 40529_2014_85_MOESM1_ESM.doc]

**Supplementary materials**

**Table S**1 Primers used for PCR

| Primer name | Sequence |
| --- | --- |
| For RACE | |
| GSP1 | 5’-ATCTTTAGCCGTAATCTGCTCGTAG-3’ |
| GSP2 | 5’-GATGATTAGAGCACCAGGAATGAAG-3’ |
| UPM-Long | 5’-CTAATACGACTCACTATAGGGCAAGCAGTGGTATCAACGCAGAGT-3’ |
| UPM-Short | 5’-CTAATACGACTCACTATAGGGC-3’ |
| NUP | 5'–AAGCAGTGGTATCAACGCAGAGT–3' |
| For full length cDNA | |
| D4HL-F | 5’- AT*CTGCAG*TGTCCCCTTTTCCTATG-3’ |
| D4HL-R | 5’- AA*GGATCC*AATGAAGGAATTGAACAAC-3’ |
| For genome walking | |
| AP1 | 5'--GTAATACGACTCACTATAGGC--3' |
| AP2 | 5'--ACTATAGGGCACGCGTGGT--3 |
| WD4HL-GSP1 | 5’- CAGAATCTACAAGTGCTTTTACTCCAG - 3’ |
| WD4HL-GSP2 | 5’ – TGTAGACTGTTCAGTGTAGTTGGCTAG - 3’ |
| For real-time quantitative PCR | |
| RSP9-F | 5’- GAGGGCCAAAACAAACTTGA -3’ |
| RSP9-R | 5’- CCCTTATGTGCCTTTGCCTA -3’ |
| d4h-like-F | 5’-GGCTCCTCATCAACCTAATCCTCAA -3’ |
| d4h-like-R | 5’-ACCAGTGTCTGTGTGTTTGCTTGT -3’ |
| For construction of GUS fusion vector | |
| D4HL-R | 5’- AT*CCATGG*AAGTGCTTTTACTCCAG -3’ |
| D4HL1K-F | 5’-TA*AAGCTT*AGCAGGCAACCTACTCTT -3’ |
| D4HL2K-F | 5’-AA*AAGCTT*CACTTATGTGGAATCTGC-3’ |
| For GUS screening | |
| GUS-S | 5'-TCACCTGCGTCAATGTAATGTTCTGC -3' |
| GUS-R | 5'-GCCGCCGACTTCGGTTTGCGGTCGCG-3' |
|  |  |


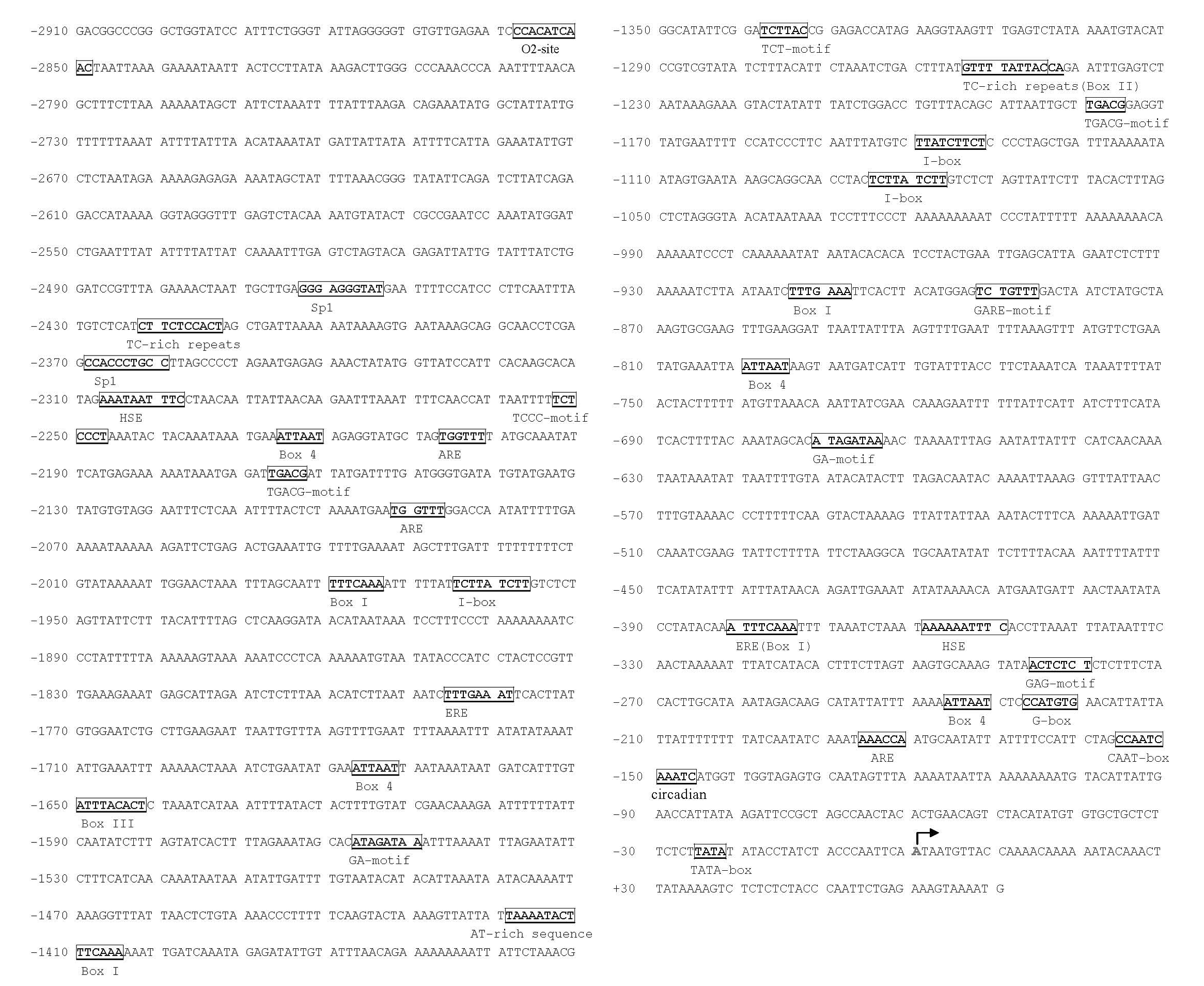
**Figure S1 Analysis of *d4h-like* promoter with PlantCare.**

Putative *cis*-elements were in square and annotated below. The transcription start site was shown with bold letter and the arrow above it indicated the transcription direction.
