# Supplementary figures and images for: An active Catharanthus roseus desacetoxyvindoline-4-hydroxylase-like gene and its transcriptional regulatory profile
Source: Bot Stud. 2014 Mar 13;55:29. doi: 10.1186/1999-3110-55-29 (PMC5430314; doi:10.1186/1999-3110-55-29)

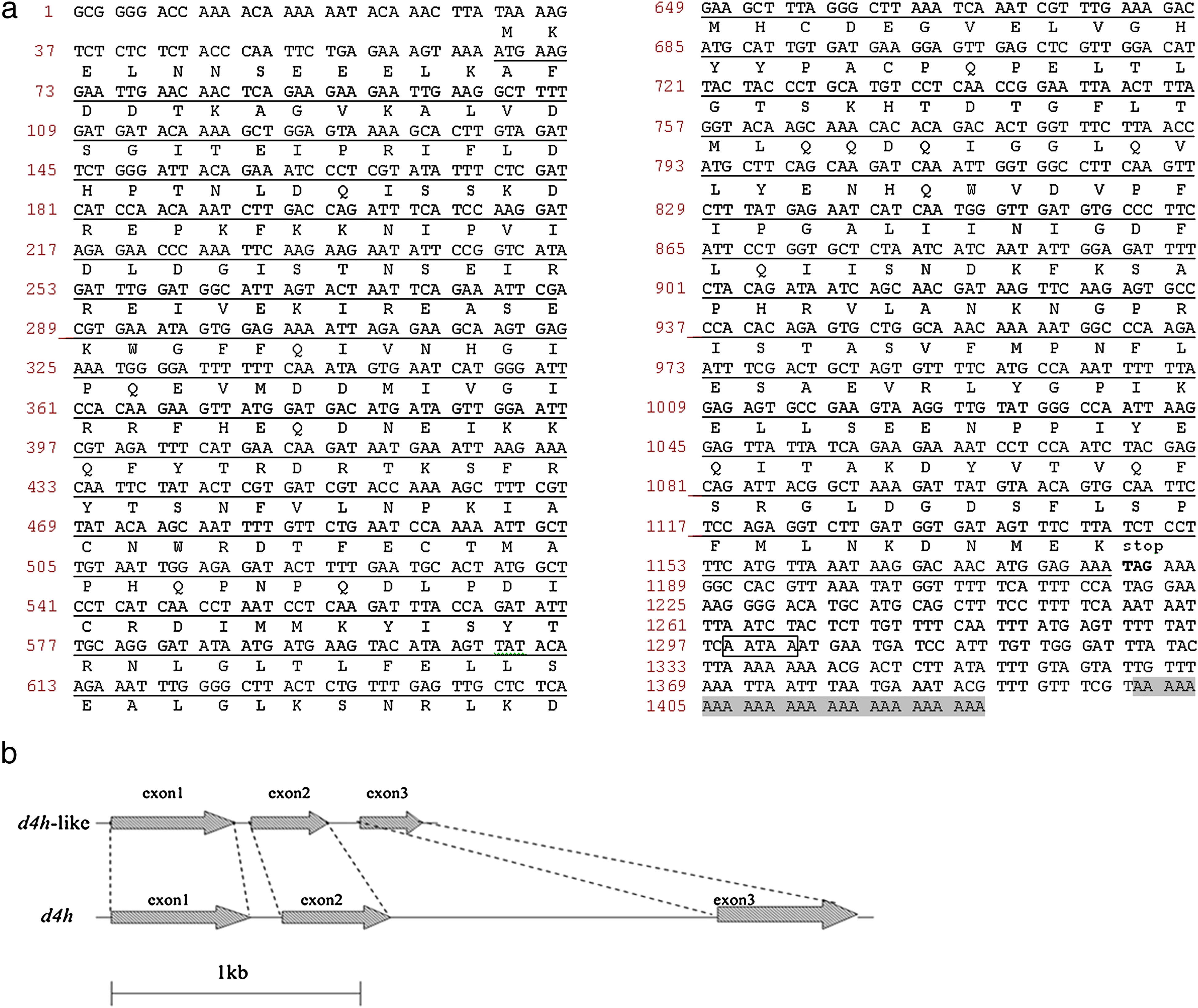

Supplement: Supplementary file 2 — Authors’ original file for figure 1 [file 40529_2014_85_MOESM2_ESM.tif]

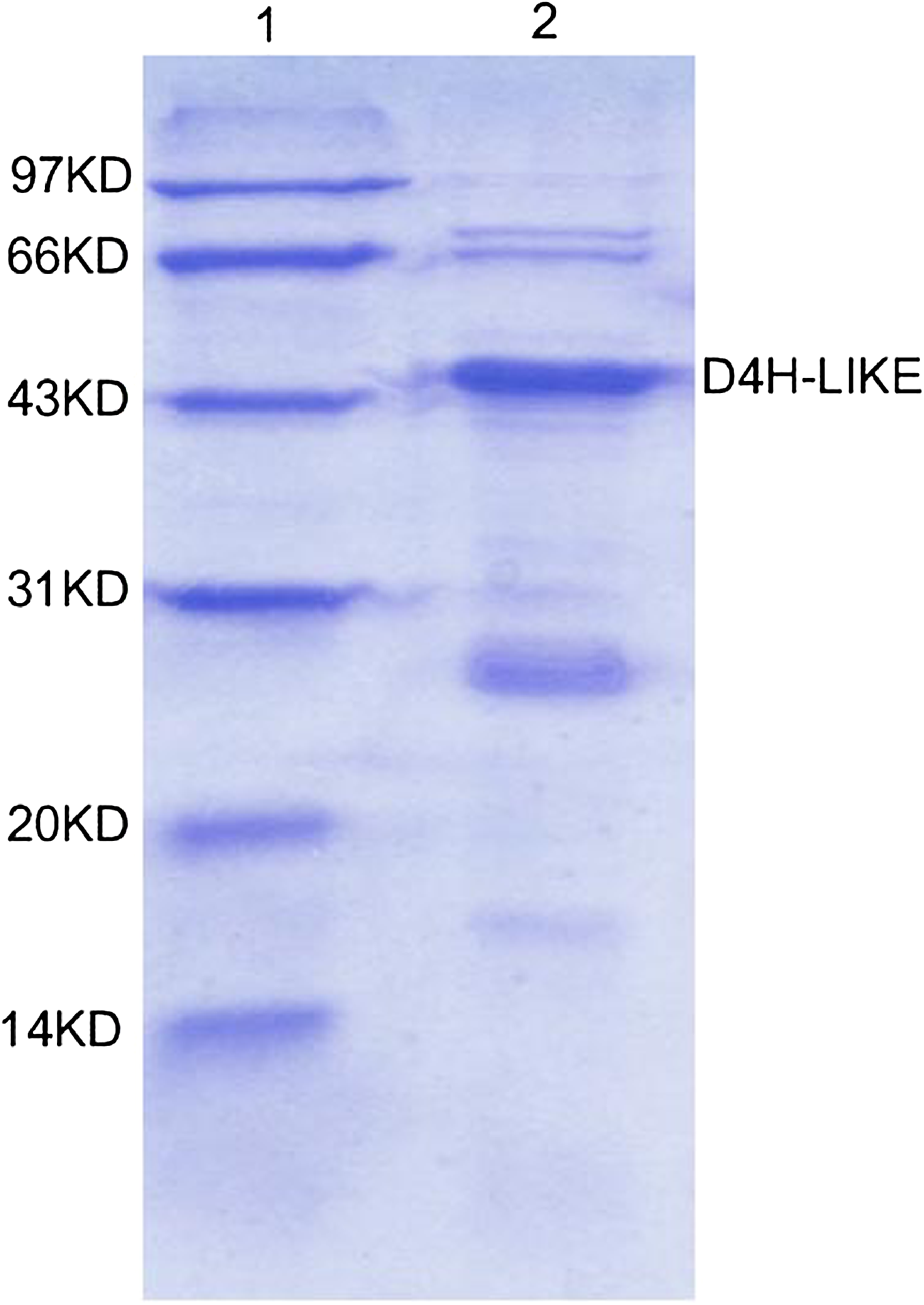

Supplement: Supplementary file 3 — Authors’ original file for figure 2 [file 40529_2014_85_MOESM3_ESM.tiff]

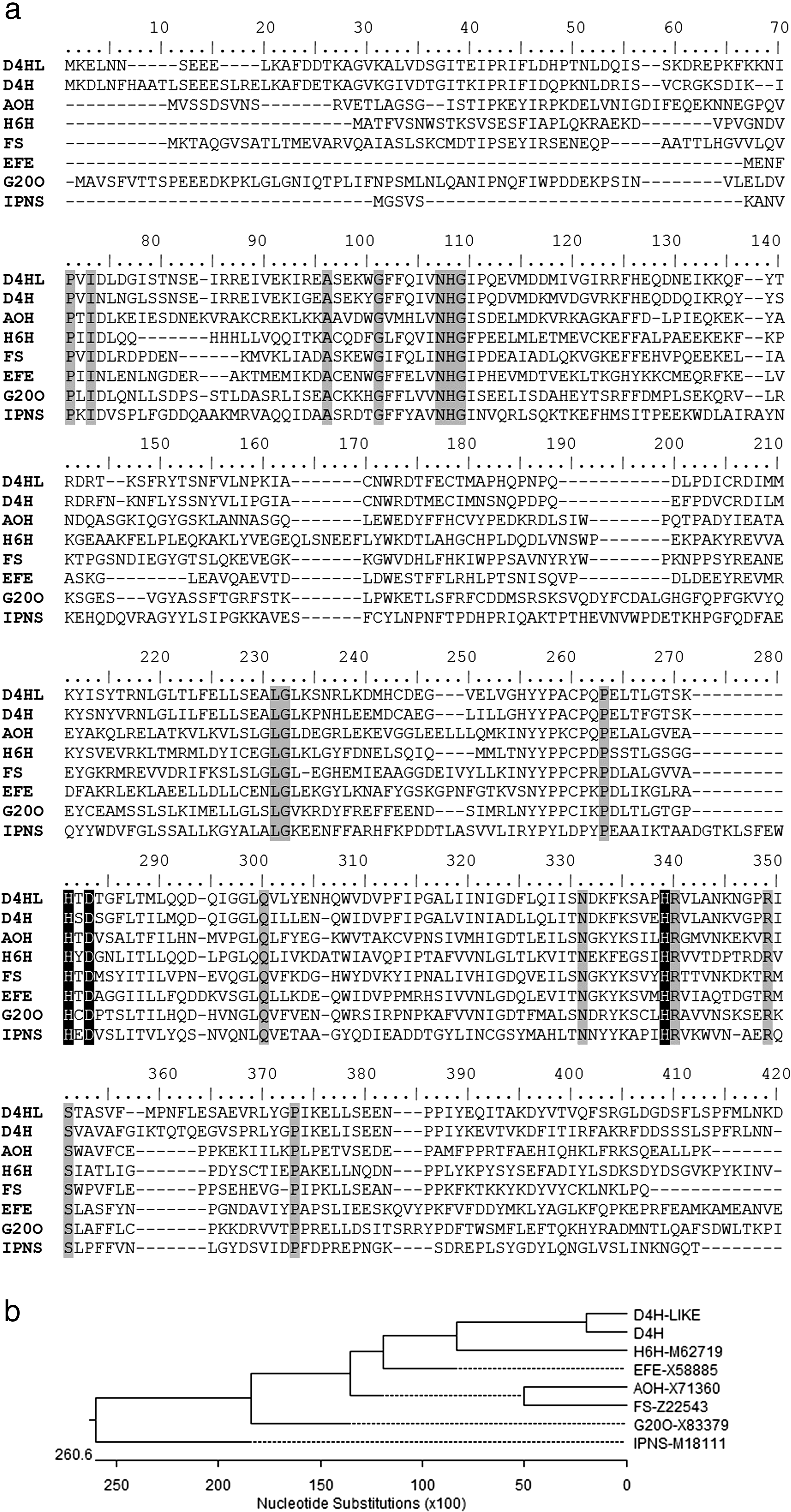

Supplement: Supplementary file 4 — Authors’ original file for figure 3 [file 40529_2014_85_MOESM4_ESM.tif]

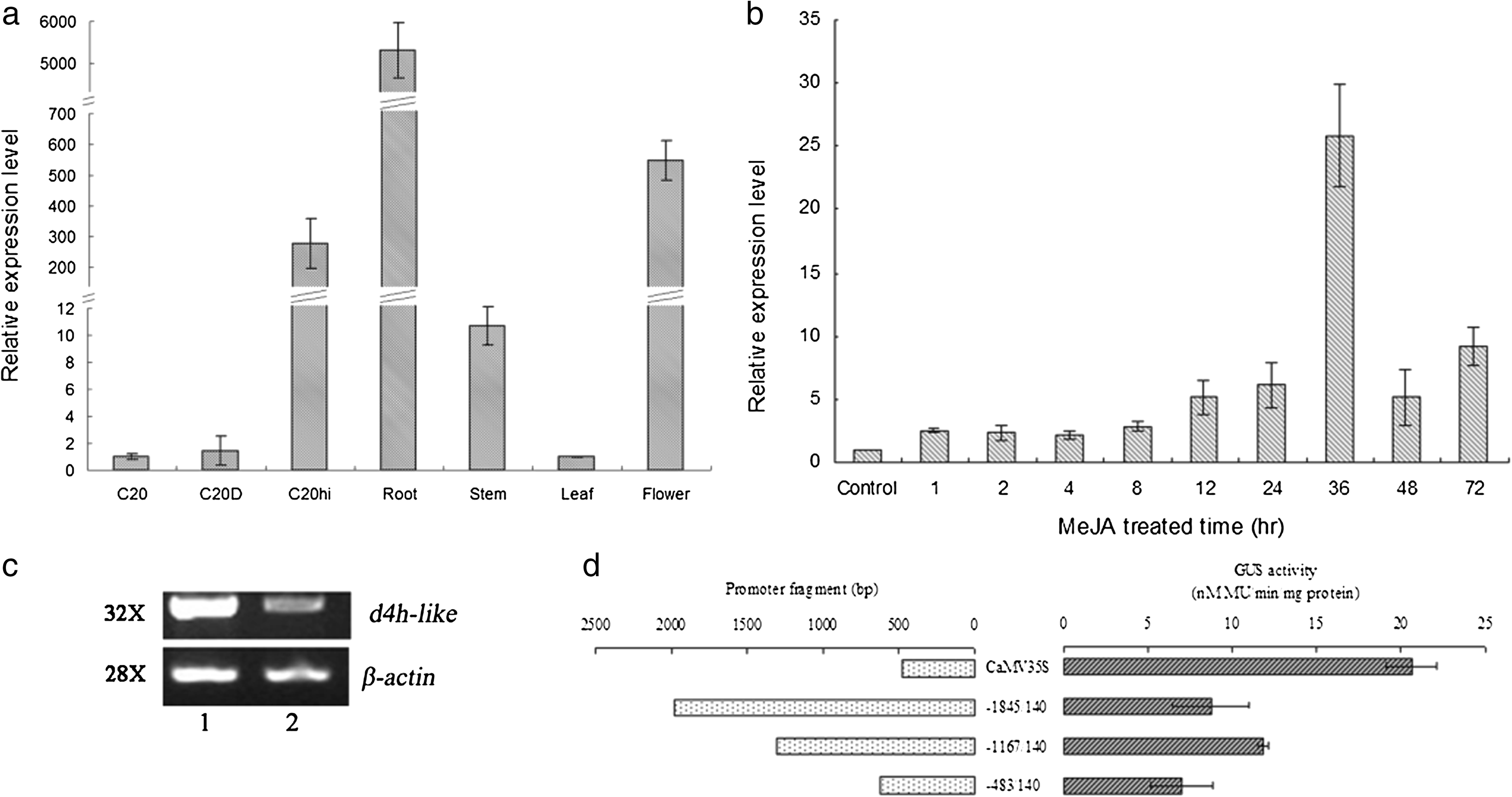

Supplement: Supplementary file 5 — Authors’ original file for figure 4 [file 40529_2014_85_MOESM5_ESM.tiff]
